# Comprehensive Electrostatic Modeling of Exposed Quantum Dots in Graphene/Hexagonal Boron Nitride Heterostructures

**DOI:** 10.3390/nano10061154

**Published:** 2020-06-12

**Authors:** Eberth A. Quezada-López, Zhehao Ge, Takashi Taniguchi, Kenji Watanabe, Frédéric Joucken, Jairo Velasco

**Affiliations:** 1 Department of Physics, University of California, Santa Cruz, CA 95064, USA; ebaqueza@ucsc.edu (E.A.Q.-L.); zge2@ucsc.edu (Z.G.); fjoucken@ucsc.edu (F.J.); 2 International Center for Materials Nanoarchitectronics National Institute for Materials Science, 1-1 Namiki, Tsukuba 305-0044, Japan; taniguchi.takashi@nims.go.jp; 3 Research Center for Functional Materials National Institute for Materials Science, 1-1 Namiki, Tsukuba 305-0044, Japan; watanabe.kenji.aml@nims.go.jp

**Keywords:** graphene, quantum dots, p-n junctions, nanoelectronics

## Abstract

Recent experimental advancements have enabled the creation of tunable localized electrostatic potentials in graphene/hexagonal boron nitride (hBN) heterostructures without concealing the graphene surface. These potentials corral graphene electrons yielding systems akin to electrostatically defined quantum dots (QDs). The spectroscopic characterization of these exposed QDs with the scanning tunneling microscope (STM) revealed intriguing resonances that are consistent with a tunneling probability of 100% across the QD walls. This effect, known as Klein tunneling, is emblematic of relativistic particles, underscoring the uniqueness of these graphene QDs. Despite the advancements with electrostatically defined graphene QDs, a complete understanding of their spectroscopic features still remains elusive. In this study, we address this lapse in knowledge by comprehensively considering the electrostatic environment of exposed graphene QDs. We then implement these considerations into tight binding calculations to enable simulations of the graphene QD local density of states. We find that the inclusion of the STM tip’s electrostatics in conjunction with that of the underlying hBN charges reproduces all of the experimentally resolved spectroscopic features. Our work provides an effective approach for modeling the electrostatics of exposed graphene QDs. The methods discussed here can be applied to other electrostatically defined QD systems that are also exposed.

## 1. Introduction

The endeavor to corral graphene’s massless Dirac fermions has led to the development of multiple techniques and novel procedures for charge carrier confinement. These techniques include the use of lithographic patterning [1,2,3,4], ultra-high vacuum chemical synthesis [5,6,7,8], controlled deposition of adatoms [9], and application of perpendicular magnetic fields [10,11,12]. However, such techniques require either complicated fabrication procedures or rely on rigid material synthesis schemes. Recently, a flexible procedure was developed to corral graphene charges that employs a scanning tunneling microscope (STM) and a graphene/hexagonal boron nitride (hBN) heterostructure [13,14]. This procedure works by using an STM tip to locally induce charge in the underlying hBN, thus creating persistent and embedded local gates within hBN. These local gates enable the corralling of electrons in an exposed circular p-n junction, which effectively behaves as a quantum dot (QD) [14]. Such QDs have been used to develop novel electronic devices such as Berry phase switches [15] and have enabled the unprecedented visualization of correlated relativistic charges under large magnetic fields [16]. Despite the rampant progress on these exposed graphene QDs, their spatially resolved spectroscopic characterization still remains not well understood.

An important commonality in existing works on exposed graphene QDs is that the influence of the STM tip has been excluded in the theoretical modeling of QD states. This is in spite of several experiments that show the STM tip itself can induce a small QD [17], and even create coulomb-like confinement [18]. In this study, we address the influence of the STM tip on exposed graphene QDs by comparing our visualization of QD states with tight-binding (TB) calculations that include the electrostatics of the STM tip and underlying charged hBN. Our results demonstrate that accounting for the influence of the STM tip is necessary to reproduce key features seen in the experimental data. Additionally, we use the insight from our study to show how the tip’s influence can be mitigated by choosing an appropriately sized QD.

## 2. Materials and Methods

**Figure 1:**
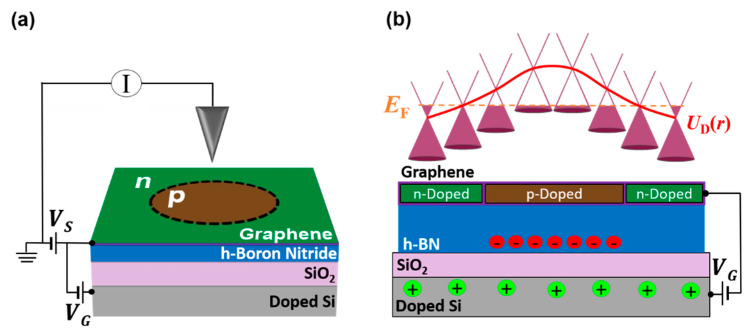
Schematic layout and potential of an exposed graphene Quantum Dot (QD). (**a**) Schematic showing the scanning tunneling microscope (STM) circuit and graphene/hexagonal boron nitride (hBN) heterostructure. The circular *p*-doped region outlines the QD created by applying a bias voltage ($V_{S}$) pulse between the tip and the graphene/hBN heterostructure while holding the back-gate voltage ($V_{G}$) constant. (**b**) Top: $U_{D}\left(r\right)$ (red curve) is the potential of the QD which is outlined by tracing along the Dirac point in each cone. Bottom: Side-view schematic of the QD in a graphene/hBN heterostructure for $V_{G}$ > 0. The application of a high electric field by the STM tip induces a localized net charge accumulation after exciting defects in hBN. Applying $V_{G}$ with opposite polarity to charges in hBN induces the spatial variation in doping on graphene which forms the QD boundaries.

The experiments we present in this work were performed on heterostructures composed of a single graphene layer on a 45 nm thick hBN resting on a SiO_2_/*p*-doped Si substrate as depicted in Figure 1a. Both graphene and hBN were mechanically exfoliated from bulk crystals. The graphene/hBN heterostructure was assembled using a standard polymer-based transfer technique [19]. Following this assembly, the surface of graphene was cleared of debris and polymer residue using a Cypher S atomic force microscope from Asylum (High Wombe, UK) while in contact mode [20]. We perform this step to ensure the absence of contaminants that could affect the electronic properties of our QDs. The creation and characterization of graphene QDs were performed with a commercial low-temperature STM (Erligheim, Germany) from Createc operating at 4.8 K.

Figure 1a shows a schematic of the STM circuit used. A sample bias ($V_{S}$) is applied between graphene and the grounded STM tip to incite a tunneling current between them and enable probing of electronic states. The back-gate ($V_{G}$) connected to the *p*-doped Si layer is used to remotely tune graphene’s Fermi level ($E_{F}$). We use these control voltages to create a graphene QD by applying a $V_{S}$ pulse between graphene and the STM tip while maintaining $V_{G}$ at a constant value [13,14,15,16,21]. During the application of this pulse, defects in hBN underneath the tip become ionized with opposite polarity to $V_{G}$. The end result of this process is depicted in Figure 1b for the case where $V_{G}>0$. At a fixed positive value of $V_{G}$, graphene becomes globally *n*-doped except for the circular region where the pulse was applied. In this region, an excess of negative charges embedded in the hBN acts as a local back-gate that *p*-dopes graphene. The resulting spatial variation of the charge neutrality point (also known as the Dirac point in graphene) with respect to $E_{F}$ (dashed orange line) gives rise to an electrostatic potential $U_{D}\left(r\right)$. A profile of this electrostatic potential is outlined in Figure 1b (red curve) and specifies the boundary of the exposed graphene QD.

## 3. Results

**Figure 2:**
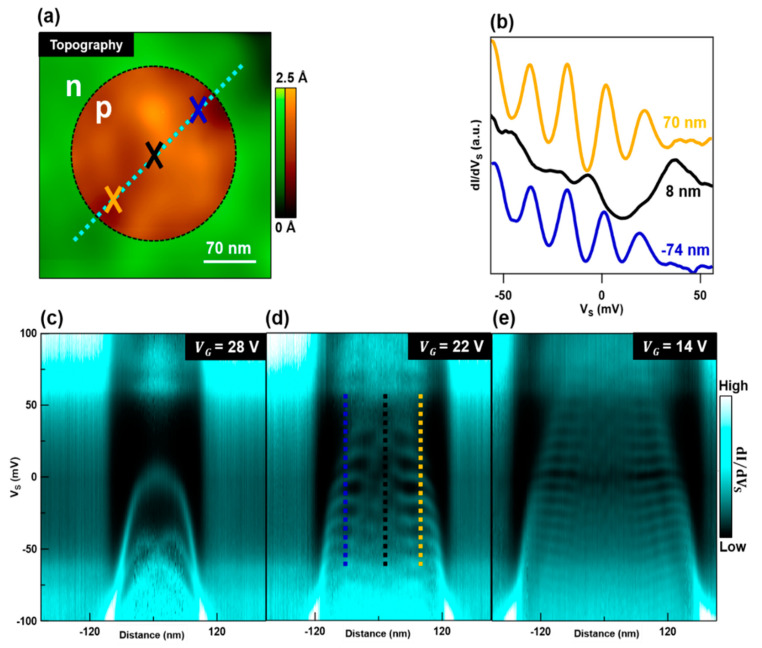
Scanning Tunneling Spectroscopy (STS) of an exposed graphene QD. (**a**) Topography of graphene acquired with the scanning tunneling microscope. Regions where graphene is *n* and *p*-doped are indicated with green and brown color scales, respectively. (**b**) Differential conductance spectra ($dI/dV_{S}$) obtained at the corresponding crosses indicated in (**a**). Each spectrum is offset for clarity and was taken with $V_{G}$ = 22 V. (**c**–**e**) Spatial dependence of $dI/dV_{S}$ spectra with different $V_{G}$, which is indicated in each panel. These images map the spatial dependence of graphene QD states obtained along the dashed cyan line in (**a**). Dashed vertical lines in (**d**) correspond to spectra displayed in (**b**). Tunneling parameters: $V_{S}$ = −0.1 V, I = 1 nA, $V_{ac}$ = 2 mV.

To map and visualize the electronic properties of the states in the exposed graphene QD, we use scanning tunneling microscopy (STM) and spectroscopy (STS). Figure 2a shows an STM topographic map of graphene’s ultra-flat surface after the creation of a QD. For the case where $V_{G}>0$, the approximate regions where graphene is *p* and *n*-doped are colored brown and green, respectively. With STS we can obtain the differential conductance ($dI/dV_{S}$) at a specific point on graphene underneath the STM tip. This $dI/dV_{S}$ signal is proportional to graphene’s local density of states (LDOS) [22]. By performing this measurement at different points within the QD, we reveal the coarse spatial dependence of the QD states. In Figure 2b we plot $dI/dV_{S}$ as a function of $V_{S}$ taken at the center (black curve) and boundaries (blue and orange curves) of the QD at points corresponding to the colored crosses in Figure 2a. These curves clearly display differences between the signals recovered at the center and edges of the QD. At the edges, the $dI/dV_{S}$ curves have prominent peaks while at the center, these peaks are suppressed and broadened.

To attain a more comprehensive understanding of the spatial dependence of the exposed graphene QD’s LDOS, we obtain $dI/dV_{S}\left(V_{S}\right)$ curves at multiple points along the dashed cyan line in Figure 2a. Figure 2c–e show the compiled $dI/dV_{S}\left(V_{S}\right)$ curves plotted as a function of distance, where the origin is defined at the center of the graphene QD. Additionally, each of these image plots are taken at different values of $V_{G}$, which offsets the global graphene doping. Following the schematic in Figure 1b, as $V_{G}$ changes, $E_{F}$ also changes relative to the QD’s potential ($U_{D}\left(r\right)$). For Figure 2c, $E_{F}$ is near the top of the potential $U_{D}\left(r\right)$, which creates a shallow QD. From the image plots in Figure 2d,e, it is apparent that as $V_{G}$ decreases (hole density increases), the QD gains depth and width as the difference between $E_{F}$ and $U_{D}\left(r\right)$ increases.

The patterns and features observed in Figure 2b–e can be explained by considering the behavior of massless Dirac fermions corralled within a circular and harmonic electrostatic potential. Due to Klein tunneling, a p-n junction on graphene perfectly transmits quasiparticles at normal incidence to the junction but reflects them at larger incident angles [23,24,25]. Therefore, in a circular potential, electrons with high angular momenta have oblique incidence with the barrier and become internally reflected. This leads to charge carrier trapping and the formation of resonant states [26,27,28,29,30] with pronounced intensities near the boundary of the circular potential, in agreement with Figure 2b. Additionally, in Figure 2b we see evidence of the internal reflection due to Klein tunneling. This can be seen in the manifestation of differing peak widths between the $dI/dV_{S}$ curve taken at the QD’s center and curves taken at the QD’s edges. As electrons with high angular momenta get trapped near the edges, these states exhibit longer trapping times and thus narrower spectroscopic peaks [14,17]. Moreover, the bright nodal features in Figs 2c–e can be attributed to the eigenstates of the exposed graphene QD [14]. The profile of the confinement potential in this QD is parabolic, akin to that of a harmonic oscillator. However, unlike Schrödinger fermions in a harmonic potential, these nodal patterns are unevenly spaced in energy. Instead, the nodal patterns formed by these massless Dirac fermions become more closely packed as $V_{S}$ decreases (see. Figure 2d) [14,17,28,29,30].

In addition to the well understood features described above, there are some features that lack explanation. For example, all three plots in Figure 2c–e show a bright skirt-like feature around the edges of the QD (~$V_{S}$ = −100 mV). A clear downward bending of the QD states is also visible near the QD boundaries for all values of $V_{G}$. This bending effect is particularly pronounced in Figure 2c, where the strong distortion of states creates an envelope-like feature. As we will soon show, these features can be reproduced after comprehensive consideration of the QD electrostatic environment.

## 4. Discussion

To study the effect of the STM tip on exposed graphene QDs, we use simplified electrostatics and a numerical tight binding model. We first discuss our considerations for the electrostatics. In our experiments, spatial variations in doping across graphene originate from localized hBN defect charges (see Figure 1b) and inadvertent gating from the STM tip. To first approximation, the localized hBN defect charges will create a fixed spatially varying doping profile. On the other hand, the STM tip will create a mobile doping profile that changes with the STM tip’s position. An exact solution for the charge density in graphene would require inaccessible experimental parameters such as the spatial distribution of hBN defect charges as well as the in-situ STM tip’s geometry. To circumvent these difficulties we proceed by making a set of simple approximations for the doping profiles due to the hBN defects and the STM tip.

**Figure 3:**
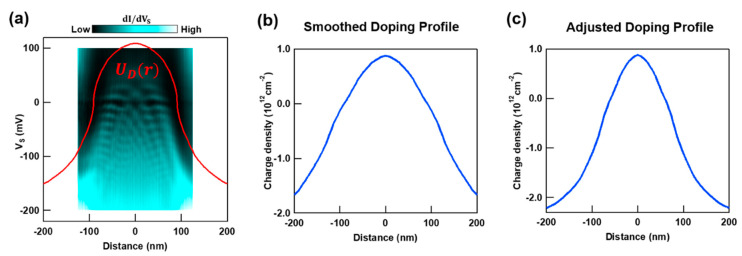
Charge density profile induced on graphene by charged hBN defects. (**a**) Experimentally approximated QD potential profile. The underlying two-dimensional (2D) plot shows the spatial dependence of QD states (similar to Figure 2c–e). The red line (same as $U_{D}\left(r\right)$ in Figure 1b) represents the extracted potential profile for the graphene QD. (**b**) QD’s charge density profile converted and smoothed from the extracted potential profile in (**a**). (**c**) QD’s charge density profile used in our tight-binding (TB) calculation after adjustment. This profile’s *x*-axis is 65% of the profile in (**b**).

We first focus on the doping profile due to charged defects in the underlying hBN. The potential profile of a graphene QD can be extracted by tracking the spatial evolution of the region with reduced $dI/dV_{S}$ intensity. This region corresponds to the spatially varying Dirac point and can be seen in measurements similar to those shown in Figure 2c–e. Figure 3a shows an example of an extracted potential profile $U_{D}\left(r\right)$ (red curve). This $U_{D}\left(r\right)$ is then converted to a doping profile n(r) by the relation n(r)= sgn$\left[U_{D}\left(r\right)\right]*\frac{U_{D}{\left(r\right)}^{2}}{\hbar^{2}v_{F}^{2}\pi }$; with $v_{F}=1\times 10^{6}\;m/s$, where $U_{D}\left(r\right)$ replaces the energy term [31]. The resulting plot after smoothing is shown in Figure 3b. We note that the STM data in Figure 3a, which is the source of our estimate for the hBN defect potential, necessarily include the effect of the STM tip. To remove this effect so that we may treat it separately, we preemptively reduce the lateral extent of the potential profile in Figure 3b to 65% while leaving the energy scale unchanged. The resulting adjusted charge density profile is shown in Figure 3c.

After obtaining the induced charge density profile on graphene due to the charged hBN defects alone, we proceed to estimate the induced charge density due to the STM tip. In our experiments, we use a tip made of tungsten. Since tungsten has a different work function than graphene, there is a finite work function mismatch (ΔΦ) between the STM tip and graphene. In the tunneling regime, the STM tip remains at a distance ~7.5 Å from the graphene surface. Because of the finite ΔΦ, there is a shift of the graphene bands even when $V_{S}$ = 0 and $V_{G}$ = 0. For $\left|\Delta \Phi \right|\gg |V_{S}|$, the polarity and intensity of the tip induced doping is dominated by ΔΦ. Therefore, in this regime we can acquire an estimate for the doping profile induced by the STM tip by obtaining an approximation of ΔΦ.

**Figure 4:**
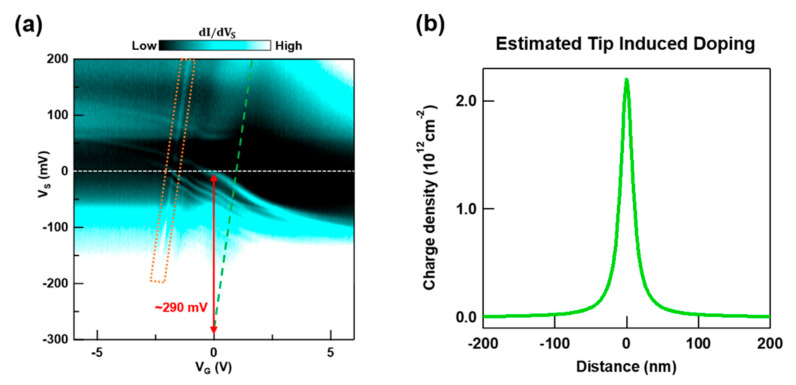
Determination of the charge density profile induced by the STM tip on graphene. (**a**) Experimentally determined $dI/dV_{S}\left(V_{G},V_{S}\right)$ on pristine graphene. This two-dimensional (2D) plot is used to determine the magnitude of the STM tip’s band shifting effect on graphene. The inverted “S” shaped features correspond to direct tunneling into tip-induced confined states. Diagonal features correspond to the charging of spectroscopic features as they coincide with $E_{F}$. The dashed orange box encloses a charging feature due to a tip-induced state and the green dashed line outlines a $dI/dV_{S}$ suppression belonging to graphene’s charge neutrality point (CNP). The dashed white line indicates where $V_{S}=0$. Tunneling parameters: $V_{S}$ = −0.2 V, I = 1 nA, $V_{ac}$ = 2 mV. (**b**) Charge density profile induced on graphene by the STM tip. The induced charge density intensity is extracted from the extrapolation in (**a**). The shape of this density profile is determined by assuming the tip to be a charged sphere with a radius of 80 nm.

To get an estimate of ΔΦ and ultimately the electrostatic effect of the STM tip, we measure and plot the dependence of $dI/dV_{S}$ as function of $V_{S}\;\mathrm{and}\;V_{G}$ on pristine graphene prior to creating a QD (see Figure 4a). The resulting two-dimensional (2D) image plot displays changes in the tunneling current between the tip and graphene as we vary their relative band alignments. In Figure 4a we observe several confined states appearing as a result of the STM tip’s local top gating effect, which were previously reported by Y. Zhao et al. [17]. As we vary $V_{S}$ for different values of $V_{G}$ we obtain a $dI/dV_{S}$ signal that includes the contribution from two channels during the tunneling process. This is a common occurrence for low dimensional systems with low charge density [32,33]. The first channel corresponds to the differential tunneling current between states of the STM tip and states in graphene at an energy determined by the sample bias. Since the horizontal axis $V_{G}$ roughly indicates changes in n and because $E_{F}\propto$ sgn$\left[n\right]\times \sqrt{\left|n\right|}$ for graphene, tip-induced states exhibit inverted “S”-shape fans in this channel. To obtain an estimate for ΔΦ, however, we focus on the second channel. This channel corresponds to the differential tunneling current between the topmost filled states in graphene and states in the STM tip. Consequently, signal traces in this channel will manifest as lines along which graphene retains a constant charge density n [21,34]. One such line appears in Figure 4a enclosed by an orange box. This feature results from the charging of a tip-induced confined state as it aligns with $E_{F}$.

After identifying the origin of the tunneling features in Figure 4a for different band alignment configurations, we are ready to obtain an estimate for ΔΦ. First, we note that for pristine graphene and for ΔΦ=0, graphene’s charge neutrality (CNP) point will cross the Fermi level at $V_{S}=0$ mV and $V_{G}=0\;V$ in a $dI/dV_{S}\;\left(V_{G},V_{S}\right)$ plot. However, because ΔΦ≠0, graphene’s CNP will cross the Fermi level at nonzero $V_{G}$ and $V_{S}$ values. To this end, we find the value of $V_{S}$ at which graphene’s charge neutrality point (CNP) crosses $E_{F}$ at $V_{G}$ = 0. With this $V_{S}$ value we can estimate the STM tip’s top gating effect due to ΔΦ on the surface of graphene in the absence of gating from below. We follow the slope of the charging feature inside the orange box and trace a green dashed line with the same slope along the furthest dark fringe on the right (see Figure 4a). The suppression of $dI/dV_{S}$ along this dark fringe indicates where graphene’s CNP crosses $E_{F}$. Finally, we extend this green dashed line downward and find that it crosses $V_{G}$ = 0 at $V_{S}\approx$ −290 mV. We note that this value for $V_{S}$ is greater in magnitude than the $V_{S}$ range in our measurements (−100 m $<V_{S}<$100 mV). Thus, we can reasonably assume that the effect of ΔΦ dominates within our experimental $V_{S}$ range used to map the QD states.

With an estimate for the graphene band shift due to ΔΦ between the STM tip and graphene we can approximate the profile for the charge induced on graphene by the STM tip. If we assume the STM tip’s apex and graphene act as a parallel plate capacitor with a 7.5 Å separation, then the $V_{S}\approx$ −290 mV offset corresponds to a maximum tip induced charge density of ~ 2.14$\times 10^{12}\;{\mathrm{cm}}^{-2}$. We calculated the shape of the tip’s doping profile by using a standard Poisson solver; where the tip is modeled by a charged sphere with an 80 nm radius that is placed 7.5 Å away from a metal surface. The tip radius and distance to graphene that we used are both consistent with values found in the literature [18,35,36]. With this analysis we acquire the tip induced doping profile shown in Figure 4b.

**Figure 5:**
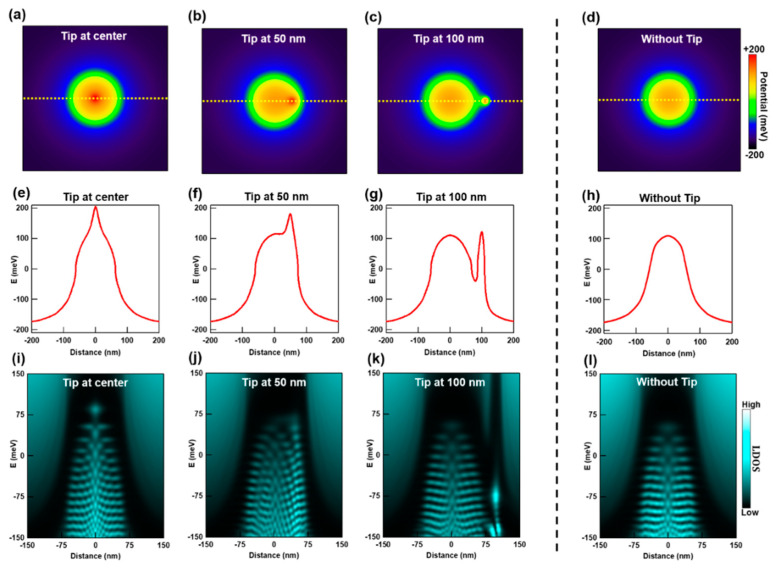
Simulation of the local density of states (LDOS) of an exposed graphene QD with an STM tip at a fixed position. (**a**–**c**) Spatial 2D map of graphene’s Dirac point energy with respect to $E_{F}$ after adding the contributions from hBN defects (Figure 3c) and the STM tip (Figure 4b). The STM tip’s location varies for each of these maps. (**d**) Spatial 2D map of the graphene Dirac point energy with respect to $E_{F}$ determined by only considering the contribution from hBN defects (Figure 3c). (**e**–**h**) Respective potential profile line cuts along the yellow dashed lines in (**a**–**d**). (**i**–**l**) Calculated LDOS distributions for each corresponding potential map. These simulations reveal the effect of the STM tip on the QD states for tip positions placed at different distances from the QD’s center.

We obtained approximations for the charge density profiles induced on graphene due to charged defects in the underlying hBN and the STM tip. We proceed by adding these contributions to obtain a potential profile resulting from the summation of the induced charge densities. After adding the charge densities induced on graphene from Figure 3d and Figure 4b, we convert the resulting charge density n(r) into a potential profile $U_{D}\left(r\right)$; where $U_{D}\left(r\right)=$ sgn$\left[n\left(r\right)\right]*\hbar v_{F}\sqrt{\pi \left|n\left(r\right)\right|}$. Figure 5a–c shows 2D maps of the potentials resulting from the cumulative charge densities of the hBN defects and STM tip. The color scale corresponds to the potential value; where red and blue indicate high and low values, respectively. Notably, each of the potential maps differ because the position of the tip changes between them. As a comparison, we also show a 2D potential map without the effect of the STM tip (Figure 5d). When the STM tip is at the center (Figure 5a) or 50 nm away from the center (Figure 5b), the potential map has the highest value at the STM tip’s location, as indicated by the red dot at the corresponding locations. Additionally, we plot line cuts of the potential maps in Figure 5e–g. Here the QD’s potential reveals a distorted profile with a prominent peak. For maps with the STM tip 100 nm away from the center (Figure 5c), the potential profile has two separate peaks (Figure 5g); effectively becoming an asymmetric double QD system.

We now discuss our numerical TB calculations, which use the potential profiles from Figure 5a–d. These calculations allow us to simulate the QD’s LDOS in the presence of a fixed STM tip. Figure 5i shows the calculated LDOS distribution of a graphene QD when the STM tip is fixed at the center. In this image we note several distinct nodes that correspond to graphene QD states [26,27,28,29,30]. In Figure 5j,k we show the calculated LDOS distributions when the STM tip is fixed 50 nm and 100 nm away from the QD’s center. As a comparison we also show the calculated LDOS of a QD that excludes the effect of the STM tip (Figure 5l). When the tip is fixed at the QD’s center (Figure 5i) or 50 nm away from the center (Figure 5j), several new states with higher LDOS appear at the tip’s location. We also note that the LDOS distribution and intensities away from the tip’s location (in Figure 5i,j) are similar to the calculated LDOS that excludes the tip’s effect (Figure 5l). When the tip is fixed 100 nm away from the QD’s center, we observe a new state with a much higher LDOS intensity at the tip’s position (Figure 5k). Similar to the previous cases, the LDOS distribution and intensities away from the tip’s location remains unaffected.

**Figure 6:**
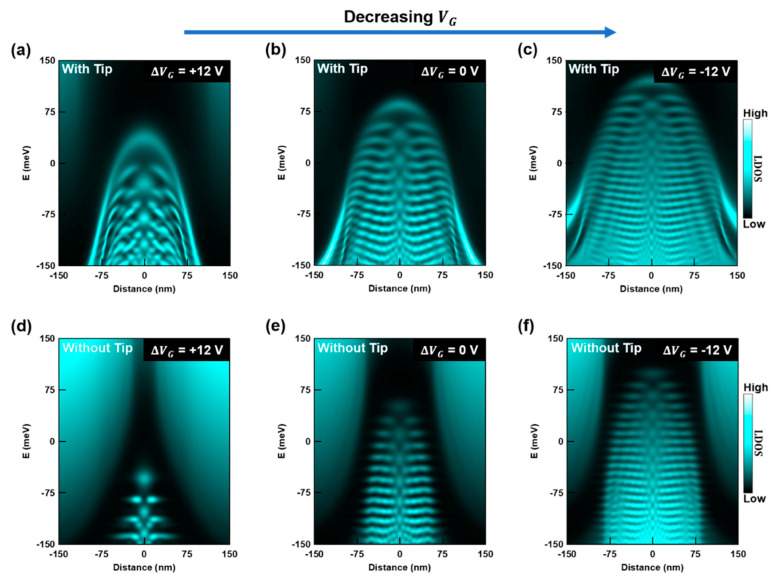
Simulation of the LDOS in a graphene QD with a moving STM tip for different $V_{G}$ configurations. (**a**–**c**) Simulated LDOS spectra along a line that crosses the center of a graphene QD. This simulation includes the influence of the STM tip. The LDOS spectra at each position is calculated with the tip fixed at that location. (**d**–**f**) Simulated LDOS spectra along a line that crosses the center of the graphene QD without considering the effect of the STM tip. The potentials induced by hBN defects used in (**a**–**c**) are the same as those used in (**d**–**f**), respectively. By comparing our results that include the effect of the STM tip (**a**–**c**) with those that exclude it (**d**–**f**), we find that a comprehensive treatment of the QD electrostatic environment is necessary to achieve agreement between theory and the experimental results from Figure 2c–e.

After demonstrating that graphene QD states are affected by an STM tip at a fixed position with our calculations, we study the case for a movable STM tip, which is more akin to our experiment. In measurements such as those shown in Figure 2c–e, each vertical array of pixels in the image corresponds to a $dI/dV_{S}$ curve acquired at the location of the STM tip. Consequently, to compare our experimental results with our simulations, we calculate the QD’s LDOS with the STM tip located at each position along a line that crosses the QD. After obtaining the LDOS distribution from each profile (similar to those in Figure 5e–g), we compile the single $dI/dV_{S}$ curves calculated specifically at the STM tip’s location for each point within the QD and along the line that crosses the QD. In Figure 6a we show the result of this compilation process.

We now consider how varying $V_{G}$ affects the QD states in our calculation. By changing $V_{G}$ the global electron and hole densities in graphene are offset. We simulate this effect by shifting the charge density profile due to the hBN defects (see Figure 3c). Specifically, this profile is shifted up to simulate an increase in hole density in graphene. Onto this shifted profile we add the unchanged STM tip’s charge density profile (Figure 4b) and perform the sequence of calculations as described for Figure 6a. Figure 6b,c show the complete results for two additional values of $V_{G}$.

To highlight the importance of the STM tip’s influence on our exposed graphene QDs, we show LDOS calculations at different $V_{G}$ values that omit the tip’s presence (Figure 6d–f). Clearly, by comparing these simulations with measurements from Figure 2c–e and the simulations in Figure 6a–c, it is evident that a tip-inclusive model achieves better agreement with our experimental results. Similar agreement can be seen with other experimental results as well [14,15,16]. Moreover, the model that omits the STM tip (Figure 6d–f) lacks several key features from our experiment. For example, the deflection of states near the edges of the QD are missing in Figs 6d–f. On the other hand, the tip-inclusive model captures this feature consistently. In addition, we note that the presence of a continuous bright line that wraps around the edge of the QD’s profile for higher $V_{G}$ values is shared by the experiment (Figure 2c) and the comprehensive model (Figure 6b), but absent in the model that ignores the tip. Finally, for the graphene QD from the experiment we note that the QD states are less distorted at higher values of $V_{G}$, see for example Figure 2e. This insightful trend is also displayed in the comprehensive model indicating that the effect of the tip can be mitigated when a sufficiently large QD is achieved. 

## 5. Conclusions

In conclusion, we showed that incorporating the STM tip’s electrostatics in conjunction with that of the underlying hBN charges enables a more complete understanding of the experimental spectroscopic features of exposed graphene QDs. We compared experimental STM data obtained on graphene QDs with simulations that include the tip-induced potential as well as with simulations that neglect this potential. The agreement between experiments and simulations is greater when the simulations include the influence of the tip. In particular, the experimentally observed bright envelope of the potential and the deflection of states close to the QD edge are only reproduced when the tip-induced potential is included. Our results highlight the importance of considering the effect of the STM tip when interpreting spectroscopic characterization of exposed graphene QD states. Our analysis also reveals the intriguing possibility of studying the interplay between states confined by the potential due to hBN defects and the potential due to the STM tip (see Figure 5k). Studies that seek to reduce such interplay may use insights from our simulations to mitigate the tip’s effect by tuning $V_{G}$. Additionally, the interaction between these two QDs could potentially be used to emulate relativistic molecular behavior or other complex coupled QD systems [37].
